# Assessment of the Bacteriocinogenic Potential of Marine Bacteria Reveals Lichenicidin Production by Seaweed-Derived *Bacillus* spp. 

**DOI:** 10.3390/md10102280

**Published:** 2012-10-18

**Authors:** Maria Luz Prieto, Laurie O’Sullivan, Shiau Pin Tan, Peter McLoughlin, Helen Hughes, Paula M. O’Connor, Paul D. Cotter, Peadar G. Lawlor, Gillian E. Gardiner

**Affiliations:** 1 Eco-Innovation Research Centre, Department of Chemical and Life Sciences, Waterford Institute of Technology, Waterford, Ireland; Email: 20038355@mail.wit.ie (M.L.P.); losullivan@wit.ie (L.O.S.); 20039108@mail.wit.ie (S.P.T.); pmcloughlin@wit.ie (P.M.); hhughes@wit.ie (H.H.); 2 Food Biosciences Department, Teagasc Food Research Centre, Teagasc, Moorepark, Fermoy, Co. Cork, Ireland; Email: paula.oconnor@teagasc.ie (P.M.O.C.); paul.cotter@teagasc.ie (P.D.C.); 3 Alimentary Pharmabiotic Centre, University College Cork, Co. Cork, Ireland; 4 Pig Development Department, Animal and Grassland Research and Innovation Centre, Teagasc, Moorepark, Fermoy, Co. Cork, Ireland; Email: peadar.lawlor@teagasc.ie

**Keywords:** antimicrobial, bacteriocin, sea, *Bacilluslicheniformis*

## Abstract

The objectives of this study were (1) to assess the bacteriocinogenic potential of bacteria derived mainly from seaweed, but also sand and seawater, (2) to identify at least some of the bacteriocins produced, if any and (3) to determine if they are unique to the marine environment and/or novel. Fifteen *Bacillus licheniformis* or *pumilus* isolates with antimicrobial activity against at least one of the indicator bacteria used were recovered. Some, at least, of the antimicrobials produced were bacteriocins, as they were proteinaceous and the producers displayed immunity. Screening with PCR primers for known *Bacillus* bacteriocins revealed that three seaweed-derived *Bacillus licheniformis* harbored the *bli04127* gene which encodes one of the peptides of the two-peptide lantibiotic lichenicidin. Production of both lichenicidin peptides was then confirmed by mass spectrometry. This is the first definitive proof of bacteriocin production by seaweed-derived bacteria. The authors acknowledge that the bacteriocin produced has previously been discovered and is not unique to the marine environment. However, the other marine isolates likely produce novel bacteriocins, as none harboured genes for known *Bacillus* bacteriocins.

## 1. Introduction

Due to the emergence of antibiotic-resistant bacteria and the increased incidence of food-borne disease there is a demand for novel antimicrobials for clinical, veterinary and food applications. Bacterial metabolites encompass a wide range of substances with diverse biological functions, including antibacterial, antiviral or antifungal activity, and as a result are often the focus of screening to identify novel antimicrobial agents. In the past, many antimicrobial compounds have been isolated from terrestrial microorganisms [1]. However, the oceans also contain a huge diversity of microbial populations, many of which are still relatively uncharacterized and therefore, represent a potentially enormous untapped resource [1]. The rationale for mining marine microorganisms for antimicrobial production is based on the fact that microorganisms that produce antimicrobial defense compounds have an advantage when competing for space and nutrients on the surfaces of marine macroorganisms [2]. Indeed, the potential of marine bacteria as antimicrobial producers has already been well documented and in some cases the responsible compounds have been identified [1]. 

Bacterially produced antimicrobial compounds are divided into two main groups; non-ribosomal secondary metabolites and ribosomally synthesized proteins/peptides, such as bacteriocins [3]. Bacteriocins are defined as “bacterially produced, small, heat-stable peptides that are active against other bacteria and to which the producer has a specific immunity mechanism” [4]. They are the most abundant and diverse of the bacterially produced antimicrobials [5]. Bacteriocins produced by lactic acid bacteria (LAB) are the most studied, followed by those produced by *Bacillus* spp. [6]. Due to their antimicrobial potency, many *Bacillus*-associated bacteriocins have considerable potential with respect to food, agricultural and pharmaceutical applications [3].

Marine bacteria have been shown to produce a number of bacteriocin-like substances [7]. However, few bacteriocins of marine origin have been fully characterized and identified to date [7] and there is no definitive proof of bacteriocin production by seaweed-associated bacteria. Therefore, the objectives of this study were (1) to assess the bacteriocinogenic potential of bacteria derived mainly from seaweed, but also sand and seawater, (2) to identify at least some of the bacteriocins produced, if any and (3) to determine if they are unique to the marine environment and/or novel.

## 2. Results

### 2.1. Isolation and Characterization of Bacteriocin-Producing Marine Bacteria

Two approaches were used to isolate bacteriocin-producing marine bacteria from a range of seaweeds, as well as one sample each of sand and seawater. The first yielded a total of 303 isolates, which were selected based on colony morphology and subsequently screened for antimicrobial activity against *Staphylococcus aureus*, *Salmonella* Typhimurium, *Listeria innocua*, *Escherichia coli* and *Bacillus subtilis*. Only seven of these (2.3%) displayed consistent activity against at least one of the target strains and were selected for further analysis. Using the second approach, more than 6000 bacterial colonies were screened against the same targets using a high throughput, combined isolation and antimicrobial detection assay. This led to the recovery of eight isolates (0.1%) with confirmed antimicrobial activity against at least one indicator organism. Half of these were generated by heating the samples prior to plating to select for spore-forming bacteria. To establish if the antimicrobial agents were released into the culture supernatant and to determine the inhibitory spectra, activity of the cell-free supernatants (CFS) was tested against the initial indicator strains as well as *Listeria monocytogenes*, *Lactococcus lactis*, *Lactobacillus bulgaricus*, *Enterococcus faecium*, *Enterococcus faecalis*, *Pseudomonas aeruginosa*, methicillin-resistant *S. aureus* (MRSA), *Cronobacter sakazakii* and *Clostridium difficile*. In total, the CFS of all 15 isolates displayed consistent antimicrobial activity against at least two indicator strains. Nine of these were isolated from marine agar (MA) (four of which were obtained by selecting for sporeformers), three from low nutrient agar (LNA), two from Actinomycete isolation agar (AIA) and one from laboratory-prepared marine agar (LPMA) (Table 1). The green seaweed *Ulva* (both *U. lactuca* and *Ulva* spp.) yielded the greatest number of antimicrobial producers (6), followed by *Polysiphonia lanosa* (3), *Fucus vesiculosus* (2), *Palmaria palmata* (1) and *Fucus serratus* (1) (Table 1).

All antimicrobial-producing isolates were Gram-positive rod-shaped, sporulating bacteria (large central spores) which were catalase positive, had variable oxidase activity and displayed a range of colony morphologies (data not shown). 16S rRNA gene sequencing revealed that the isolates were either *Bacillus pumilus* (7 isolates) or *licheniformis* (8 isolates) (Table 1; Figure S1). Comparison of PFGE fingerprints generated with *Not*I demonstrated that *B. pumilus* WIT 560 and WIT 561 were essentially the same strain (Figure S2), whereas, the remainder of the marine isolates differed from one another. These findings were confirmed by further PFGE typing using *Xba*I- and in some cases *Apa*I-digested DNA, as not all isolates were digested by the latter (data not shown). 

All 15 of the selected antimicrobial-producing marine isolates inhibited the growth of *Lc. lactis* HP and *Lb. bulgaricus* LMG 6901 when the CFS was assessed by the agar well diffusion assay (WDA) (data not shown). Moreover, 13 isolates had antimicrobial activity against *S. aureus*, 12 against *L. innocua*, 13 against *L. monocytogenes*, six against *B. subtilis* and three against *E. faecium* in the WDA and eight inhibited *E. faecalis* in the deferred antagonism assay (Table 1). Nine isolates displayed activity against MRSA, five of which (WIT 560, 561, 570, 571 and 572) produced a zone of inhibition with a radius of up to 3 mm (Table 1). All of the isolates that were active against MRSA also inhibited *S. aureus* DPC 5246. None of the CFS from any of the marine isolates inhibited any of the Gram-negative indicator bacteria tested but nine isolates displayed activity against *E. coli*, *S.* Typhimurium and/or *C. sakazakii* when assessed using the deferred antagonism assay (Table 1). WIT 560 and WIT 561 had the broadest spectra of inhibition, as they were active against 10 different indicator bacteria, both Gram-positive and -negative (*Listeria*, *S. aureus*, MRSA, *Lc. lactis*, *Lb. bulgaricus*, *E. faecalis*, *C. sakazakii*, *E. coli* and *S.* Typhimurium). Similarities between the inhibitory spectra of these isolates support our assertion that they represent the same strain of *B. pumilus*.

**Table 1:** Inhibitory spectra of antimicrobial-producing bacteria isolated from seaweeds, sand and seawater.

Isolate no. (identified by 16S rRNA gene sequencing)	Origin	Isolation medium	Culture Supernatants (Well diffusion assay) a								Culture (deferred antagonism assay) a			
				*L. innocua* WIT 361	*L. monocytogenes* WIT 041	*B. subtilis* ATCC 6633	*S. aureus* DPC 5246	MRSA W73365	*E. faecium* ATCC 19434		*E. coli* DSM 10720	*S. *Typhimurium LT2	*E. faecalis* ATCC 19433	*C. sakazakii* ATCC 12868
***B. licheniformis* WIT 562 b**	***P. lanosa***	**LPMA**		+/-	+/-	-	-	-	-		-	-	-	-
***B. licheniformis* WIT 564 b**	***Ulva spp.***	**LNA**		-	-	+	+	-	-		-	-	-	-
**B. licheniformis WIT 566 b**	***Ulva* spp.**	**MA**		+	-	+	-	-	-		-	-	-	-
*B. pumilus* WIT 560 c	*F. vesiculosus*	LNA		++	++	-	++++	+++	-		++++	++	+	+++
*B. pumilus *WIT 561 c	*F. vesiculosus*	LNA		++	++	-	+++	+++	-		++++	+++	+	++++
*B. pumilus* WIT 563	*Ulva* spp.	MA		+	+/-	-	+++	++	-		++++	+/-	+	++
*B. licheniformis* WIT 565	*Ulva* spp.	MA		++	+/-	+	+	-	-		++++	+/-	+	++
*B. licheniformis* WIT 567	Sand	MA		-	++	+	++	+	++++		-	-	-	-
*B. licheniformis* WIT 568	Seawater	MA		-	+	+	++	+	++++		-	-	-	-
B. licheniformis WIT 569	*U. lactuca*	MA ^d^		+	+	+	+++	-	++++		-	-	+	-
*B. licheniformis* WIT 570	*U. lactuca*	MA ^d^		+++	+++	-	+++	+++	-		-	-	++	-
*B. pumilus *WIT 571	*P. lanosa*	MA ^d^		++	++	-	+++	+++	-		-	-	-	-
*B. pumilus* WIT 572	*P. lanosa*	MA ^d^		+++	++	-	+++	+++	-		++++	+/-	+	+++
*B. pumilus* WIT 573	*P. palmata*	AIA		+	+	-	+++	+	-		++++	+/-	++	+/-
*B. pumilus* WIT 574	*F. serratus*	AIA		+	++	-	++	-	-		+/-	-	-	+/-
*Lc. Lactis* NZ 9700 e	NA ^f^	NA		++	-	+	++	-	++		ND	ND	ND	ND

^a^ Mean radii of zones of inhibition from triplicate assays. **+** = 0.1–1 mm, **++** = 1.1–2 mm, **+++** = 2.1–3 mm, **++++** >3 mm; - = no antimicrobial activity; +/- = variable activity. ND = not determined. All isolates were also active against *Lc. lactis* HP and *Lb. bulgaricus* LMG 6901 in the well diffusion assay (data not shown). No antimicrobial activity was observed against *Cl. difficile* ATCC 43593 or *P. aeruginosa* PA01; ^b^ Isolates subsequently shown to produce lichenicidin are grouped together and shown in bold; ^c^ Denotes isolates with identical genetic fingerprints; ^d^ Sample heated to 80 °C for 15 min prior to plating; ^e^ *Lc. lactis* NZ 9700 (nisin producer used as control); ^f^ NA = Not applicable.

### 2.2. Characterization of Antimicrobial Compounds Produced by Marine Bacteria

#### 2.2.1. Effect of Growth Medium on Antimicrobial Production

The effect of several growth media on antimicrobial production by the 15 antimicrobial-producing isolates was evaluated using the WDA. These assays revealed that activity was greatest when the isolates were cultured in marine broth, nutrient broth or *Bacillus* production medium (Table S1). 

#### 2.2.2. Cross Sensitivity Assays

Cross sensitivity assays were performed to assess the ability of the marine isolates to inhibit each other and other *Bacillus* species that produce known bacteriocins (Table S2). Because bacteria are usually resistant to the compounds they produce themselves, if a bacterial isolate is resistant to an antimicrobial produced by another strain, both are likely to produce the same compound. None of the isolates displayed antimicrobial activity against themselves, indicating that they are immune to the antimicrobial compounds they produce. *B. pumilus* WIT 560 and WIT 561 had similar inhibitory spectra against related (Table S2) and non-related (Table 1) species and did not inhibit each other. However, although *B. licheniformis* WIT 565 and WIT 566, *B. licheniformis* WIT 568 and WIT 569, and *B. pumilus* WIT 571 and WIT 572 displayed similar inhibitory spectra against closely-related species (Table S2), they differed with respect to their activity against unrelated species (Table 1). *B. licheniformis* WIT 564 and WIT 570 displayed activity against all of the other marine-derived *Bacillus* isolates. *B. licheniformis* ATCC 14580 (lichenicidin producer), *Bacillus halodurans* ATCC BAA-125D-5 (haloduracin producer), *Bacillus megaterium* 216 (megacin producer) and *Bacillus cereus* CECT 5148 (cerein producer) did not inhibit the marine-derived isolates. However, the haloduracin producer was sensitive to the antimicrobials produced by all of the marine-derived *Bacillus*, the subtilin, lichenicidin and megacin producers were sensitive to those produced by about half of the marine isolates and the cerein producer was sensitive to only one. 

#### 2.2.3. Physicochemical Characterization of Antimicrobial CFS

The effect of various proteolytic enzymes, catalase, pH and heat treatment on the activity of the antimicrobial compounds present in the CFS of the marine isolates was evaluated (Table 2). The CFS from all of the isolates lost complete or partial activity when treated with at least one proteolytic enzyme. In this respect, they compared well with the CFS of the nisin-producing strain *Lc. lactis* NZ 9700. Complete loss of activity was observed in the CFS of 10 isolates when treated with pronase E and seven were completely inactivated following treatment with proteinase K (Table 2). With the exception of the *B. pumilus* isolates WIT 563 and WIT 573, all of the marine CFS retained some, if not all, activity following incubation with catalase. In addition, the antimicrobial(s) produced by WIT 573 was fully susceptible to all of the proteolytic enzymes and the CFS of WIT 563 was completely sensitive to most and its activity was reduced by the others (Table 2). On the other hand, the antimicrobial(s) produced by *B. licheniformis* WIT 565 was unique by virtue of being resistant to all but pronase E.

**Table 2:** Effect of enzyme and heat treatment and pH on antimicrobial activity^a^ of cell-free supernatants from marine isolates.

Marine Isolate	Sensitive to ^b^	Resistant to (temperature, °C) ^c^	Resistant to(pH range) ^d^
**WIT 562** **^e^**	**Pronase E, proteinase K, pepsin, catalase, α-chymotrypsin, trypsin, protease type I**	**50 (60)**	**5–9 (2)**
**WIT 564** **^e^**	**Pronase E, proteinase K, trypsin**	**50 (60)**	**3–9 (2)**
**WIT 566** **^e^**	**Pronase E, proteinase K, catalase**	**50 (60)**	**5–9 (2, 11)**
WIT 560 ^f^	Pronase E, proteinase K, pepsin, α-chymotrypsin, trypsin, protease type I	50 (70)	5–9 (2, 11)
WIT 561 ^f^	Pronase E, proteinase K, pepsin, α-chymotrypsin, protease type I	50 (70)	2–9 (12)
WIT 563	Pronase E, proteinase K, pepsin, catalase, α-chymotrypsin, trypsin, protease type I	70 (80)	2–9 (12)
WIT 565	Pronase E	50 (60)	3–9 (2, 11)
WIT 567	Pronase E, proteinase K, pepsin, α-chymotrypsin, trypsin	90 (121)	2–9 (12)
WIT 568	Pronase E, α-chymotrypsin, trypsin, protease type I	50 (121)	3–9 (2, 12)
WIT 569	Pronase E, proteinase K, pepsin, α-chymotrypsin, trypsin	90 (121)	3–9 (2, 12)
WIT 570	Proteinase K, pepsin, α-chymotrypsin, trypsin	90 (121)	5–9 (2, 12)
WIT 571	Pronase E, proteinase K, trypsin	40 (80)	5–9 (2, 12)
WIT 572	Pronase E, proteinase K, pepsin, α-chymotrypsin, trypsin, protease type I	50 (60)	3–9 (2, 12)
WIT 573	Pronase E, proteinase K, pepsin, catalase, α-chymotrypsin, trypsin, protease type I	50 (60)	5–7 (2, 11)
WIT 574	Proteinase K, α-chymotrypsin, protease type I	50 (60)	2–9
NZ 9700 ^g^	Pronase E, proteinase K, pepsin, α-chymotrypsin, trypsin, protease type I	70 (100)	2–9 (11)

^a^ Antimicrobial activity was determined against *Lc. lactis* HP using the well diffusion assay and data are the mean of triplicate assays. ^b^ Complete (100%) loss of activity was observed when the cell-free supernatants were incubated at 37 °C for 2 h with 5 mg/mL of each of the listed enzymes, except for the underlined enzymes for which only reduced (17%–83%) activity was observed. ^c^ Maximum temperature at which 100% activity was retained. Reduced (17%–83%) activity was observed at the temperature listed in parentheses. All heat treatments were for a duration of 30 min in a heating block, except for 121 °C, which was for 15 min in an autoclave. ^d^ pH range over which 100% activity was retained. Reduced (17%–83%) activity was observed up to the pH values given in parentheses. All cell-free supernatants were incubated for 2 h at 28 °C following adjustment to pH 2, 3, 5, 7, 9, 11 or 12. ^e^ Isolates subsequently shown to produce lichenicidin are grouped together and shown in bold. ^f^ Denotes isolates with identical genetic fingerprints. ^g^ *Lc. lactis* NZ 9700 (nisin producer used as a control).

The antimicrobial activity of the CFS from all isolates remained unaffected following heating to 50 °C for 15 min (Table 2). However, the CFS of half of the isolates i.e. *B. licheniformis* WIT 562, 564, 565 and 566 and *B. pumilus* WIT 572, 573 and 574 were relatively heat sensitive, as they lost all activity at temperatures above 60 °C. Compounds produced by *B. licheniformis* WIT 567, 568, 569 and 570 were the most heat resistant, retaining 50%–83% activity even after autoclaving at 121 °C for 15 min; in comparison, the CFS from the nisin-producing strain retained only 10% activity (data not shown). The antimicrobials produced by the remaining isolates were also relatively heat resistant, retaining at least some activity at temperatures of 70–80 °C (Table 2). 

The optimum pH for the antimicrobial compounds produced by all of the marine isolates was 5 or 7; however, at least some of the antimicrobial activity of all of the CFS was retained over the pH range 2–9 (Table 2). The CFS from four of the isolates even retained full activity over this pH range, and in this respect were comparable to the nisin-producing control strain. Furthermore, the CFS from the majority of the isolates were active even at pH 11 and 12, albeit activity was reduced. 

### 2.3. Screening for Known Bacteriocin-Associated Genes

**Figure 1:**
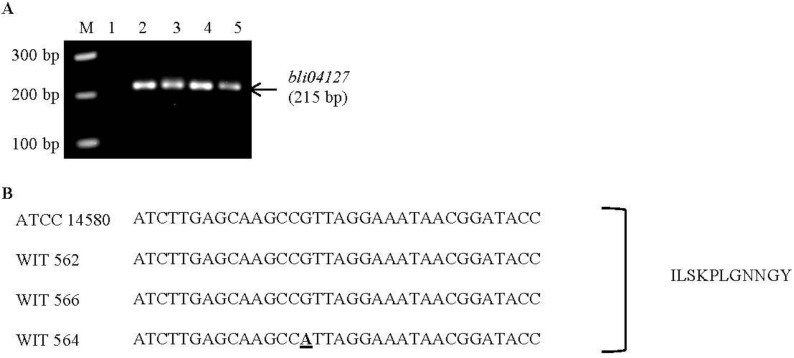
(**a**) Agarose gel electrophoresis of PCR products generated using primers specific for the *bli04127* gene which encodes the Bliα lichenicidin peptide. M: 100-bp ladder; Lane 1: water as a negative control; lane 2: *B. licheniformis* ATCC 14580 (lichenicidin-producing positive control strain); lane 3: *B. licheniformis* WIT 562; lane 4*: B. licheniformis* WIT 564; lane 5: *B. licheniformis* WIT 566. (**b**) Part of the nucleotide sequence of the *bli04127* gene which was amplified from *B. licheniformis* ATCC 14580 (lichenicidin-producing positive control strain), *B. licheniformis* WIT 562, *B. licheniformis* WIT 564 and *B. licheniformis* WIT 566. Nucleotide differences are in bold and underlined. The corresponding part of the predicted peptide sequence is also shown and was the same for all isolates. The entire *bli04127*gene was sequenced in all isolates and the entire Bliα peptide sequence predicted but only partial sequences are shown.

In order to investigate if the marine bacteria produce a previously identified bacteriocin, PCR analysis was performed using primer pairs specific for known *Bacillus* bacteriocins. Nine of these were designed in the present study and six had previously been used by others (Table 3). The marine isolates were also screened for the presence of three lantibiotic-associated genes. PCR analysis using primers designed to amplify the *bli04127* gene, which is the structural gene of the Bliα lichenicidin peptide and part of the lichenicidin gene cluster, yielded amplicons of the appropriate size when *B. licheniformis* WIT 562, 564 and 566 were tested (Figure 1a). This was in agreement with results obtained for the known lichenicidin producer, *B. licheniformis* ATCC 14580. Sequencing of the amplicons from WIT 562 and 566 revealed 100% homology to the *bli04127* gene (Figure 1b). The same was true for the ATCC 14580 strain (the known lichenicidin producer). However, analysis of the WIT 564 sequence revealed one nucleotide difference. This difference, however, does not result in any amino acid change and the amplicons from all three marine isolates, as well as the lichenicidin-producing control strain, are predicted to encode the Bliα propeptide. This confirms that the three marine isolates harbor the lichenicidin-encoding operon. All other PCR assays for other known bacteriocins failed to generate appropriately sized amplicons from any of the marine isolates. The relevant positive control strains did, however, yield amplicons of the expected size (data not shown), indicating that the PCR conditions were appropriate to amplify the genes, had they been present. These data indicate the absence of the associated bacteriocin gene clusters from the marine isolates.

**Table 3:** List of bacteriocin primers used in this study.

Bacteriocin	Gene	Sequence (5′-3′)	Size of expected product (bp)	Annealing temperature (°C)	Positive control strain ^a^	Genbank accession number of bacteriocin sequence	Reference
Subtilin	*Spa*	ACTATGAATCAATGGAAGG	370	50	*B. subtilis* ATCC 6633	M99263.1	[8]
		TTGCAGTTACAAGTTAGTG					
Subtilosin	*Sbo*	GGTTGTGCAACATGCTCGAT	300	58	*B. subtilis* ATCC 6633	AJ430547.1	[8]
		CTCAGGAAGCTGGTGAACTC					
Sublancin	*Sun*	GTGTGCTGCGTTGTGGCTACAA	230	62	*B. subtilis* 168	NC_000964.3	[8]
		TTGACGAGATACAAGCTAGTCC					
Coagulin	*CoaA*	GGTGGTAAATACTACGGTAATGGGGT	~600	66	*B. coagulans* I4	AF300457	[9]
		GTGTCTAAATTACTGGTTGATTCGT					
Mersacidin	*MrsA*	CTTAATAAGGGGGTAATAC	270	56	*B. subtilis* HIL	Z47559.1	This study
		TAGGCTGTTCCTTCTGAAGG			Y-85,54728		
Lichenicidin	*Bli04127*	GGAAATGATTCTTTCATGG	215	60	*B. licheniformis* ATCC	CP000002.3	This study
		TTAGTTACAGCTTGGCATG			14850		
Ericin A	*EriSa*	TGTCAAAGTTCGATGACTTC	171	56	*B. subtilis* A 1/3	AF233755.1	This study
		TCAGCACTTAGCAAATGTTG					
Haloduracin A1	*BH0454*	ATGGAAAATGCCTCTTGAG	191	54	*B. halodurans* ATCC	BA000004.3	This study
		TTAGTTGCAAGAAGGCATG			BAA-125D-5		
Haloduracin A2	*BH0453*	TTAGCACTGGCTTGTACACT	180	58	*B. halodurans* ATCC	BA000004.3	This study
		TTGCGTAATCCTGAATTCCG			BAA-125D-5		
Thuricin17	*TucA1, A2* & *A3*	GTAGGTCAAATGGAAACAC	589	52	*B. thuringiensis* NEB17	FJ159242.1	This study
		TTAACTTGCAGTACTAGCTC					
Thurincin H	*H1*, *H2* & *H3*	ATGGAAACACCAGTAGTACA	579	56	*B. thuringiensis* SF361	FJ977580.1	This study
		TTAACTTGCAGTACTAGCTC					
Megacin A-216	*P293A*	TTACATACCATGAGAAGCGCAT	519	66	*B. megaterium* 216	EU014074.1	This study
		CATGTTAGTGCAGTTTACCTTC					
Cerein 7B	*Cer7B*	ATAGCTGGGGTAAATGTGTTG	153	62	*B. cereus* CECT 5148	AM087432.1	This study
		AAAGTAGCTGCACCTGTAAG					
Class I-Type I	*LanC*	TAATTTAGGATWISYIMAYGG	~250	40	*Lc. lactis* NZ 9700	NA ^b^	[10]
Lantibiotic		ACCWGKIIIICCRTRRCACCA					
	*LanB*	TATGATCGAGAARYAKAWAGATATGG	~400	44	*Lc. lactis* NZ 9700	NA	[10]
		TTATTAIRCAIATGIAYDAWACT					
Class I-Type II	*LanM*	TTGCWAGWYWTGCWCATGG	330	49	*Lc. lactis* DPC 3147	NA	[11]
Lantibiotic		CCTAATGAACCRTRRYAYCA					

^a^ All positive control strains yielded PCR amplicons of the expected size. However, no positive control was used for thurincin H, as the producing strain could not be obtained; ^b^ NA = Not applicable.

### 2.4. Purification of Lichenicidin

**Figure 2:**
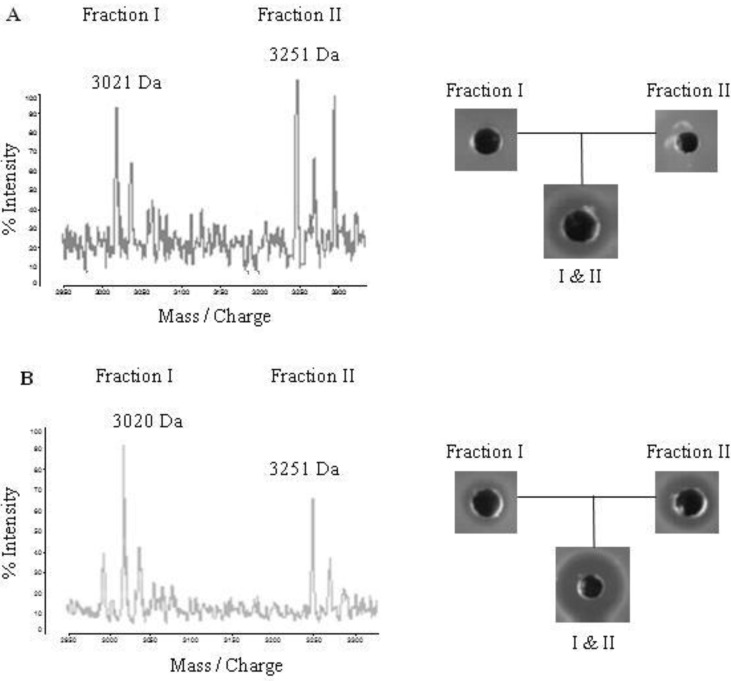
(**a**) Matrix-assisted laser desorption ionization time of flight (MALDI-TOF) mass spectrometry analysis of RP-HPLC fractions from a *B. licheniformis* WIT 564 extract, showing the Bliα and Bliβ lichenicidin peptides. Similar mass spectra were obtained for fractions extracted from *B. licheniformis* WIT 562 and WIT 566 (data not shown). Analysis of antimicrobial activity of the fractions by well diffusion assay using *Lc. lactis* HP as an indicator is also shown, demonstrating that the fractions were inactive separately but showed antimicrobial activity when combined. (**b**) MALDI-TOF mass spectrometry and antimicrobial activity of RP-HPLC fractions from the lichenicidin-producing control strain (*B. licheniformis* ATCC 14580) for comparison. In this case, HPLC fractions containing the lichenicidin peptides were active separately but activity was enhanced when they were combined.

In order to confirm that *B. licheniformis* WIT 562, 564 and 566 produce lichenicidin, the two-peptide bacteriocin was extracted from all three marine isolates, purified and compared to lichenicidin produced by *B. licheniformis* ATCC 14580. HPLC fractions of extracts from the marine-derived bacteria with masses corresponding to those previously determined for the Bliα and Bliβ lichenicidin peptides (~3021 Da and ~3251 Da, respectively [12]) did not show activity when each was tested separately (Figure 2a). However, activity was detected when the fractions were combined. This contrasted somewhat with the corresponding fractions from the positive control strain *B. licheniformis* ATCC 14580, each of which had antimicrobial activity, which was enhanced when tested in combination (Figure 2b). 

## 3. Discussion

The value of marine bacteria as a source of antimicrobials is now being recognized [1]. However, few bacteriocins of marine origin have been fully characterized and identified to date [7] and there is no definitive proof of bacteriocin production by seaweed-associated bacteria. This is the first study to assess the bacteriocinogenic potential of bacteria from Irish seaweeds. Fifteen isolates with confirmed consistent antimicrobial activity were recovered from seaweed, as well as sand and seawater. However, the recovery rate (2.3% of pure cultures screened) was lower than that reported in previous studies using a similar approach (11%–16%, [13,14]). This is perhaps not surprising and can be attributed to geographic and source variation as well as the use of different target organisms and selection criteria. The high throughput, combined isolation and antimicrobial detection assay also used facilitated more rapid screening of a larger number of marine isolates but was less successful than when previously used for intestinal samples [15]. The green seaweeds (*Ulva* spp. and *U. lactuca*) yielded the highest number of antimicrobial producers and the recovery rate from sand and seawater was also relatively high, considering that only one sample of each was analyzed. This should be borne in mind for future screening studies. All 15 antimicrobial-producing isolates recovered were spore-forming bacteria, subsequently identified as *Bacillus* spp., providing further evidence of the value of this genus as a source of antimicrobials [6]. This also suggests that specifically targeting spore-forming bacteria, an approach recently employed by Phelan *et al.* when screening sponge-associated microflora [16], may be a more favorable strategy for future studies. 

The antimicrobial compounds produced by the marine-derived *Bacillus* were active against Gram-positive (MRSA, *L. monocytogenes*, *E. faecalis*)and Gram-negative (*S.* Typhimurium, *E. coli*, *C. sakazakii*) pathogens as well as non-pathogenic indicator strains, such as *L. innocua*, *Bacillus* spp. and other *S. aureus*. Although no one isolate inhibited all of the targets tested, some were relatively broad spectrum, killing both Gram-positive and -negative bacteria. Inhibition of Gram-positives was more common, in keeping with the findings of other studies of marine bacteria [17]. The fact that the main targets are Gram-positive suggests that at least some of the compounds produced are bacteriocins, as bacteriocins usually inhibit species closely related to the producers. The fact that anti-Gram-negative activity was also detected, although not in the CFS, is another interesting, but as yet unexplored, facet of the study. The finding that all of the marine isolates were resistant to the antimicrobials they produce provides further evidence that the compounds are bacteriocins, as this is one of their distinguishing features [5]. The reduction or disappearance of activity after incubation with proteolytic enzymes also supports this. However, the fact that some of the CFS also lost activity when treated with catalase suggests the involvement of hydrogen peroxide in some cases. 

Having established that the marine isolates produce bacteriocins, the next step was to attempt to identify at least some of the compounds produced and to determine if any were novel. Cross sensitivity assays with known bacteriocin-producing strains can help to determine if bacteriocins have previously been identified. This is because, bacteriocin-producing strains are usually resistant to the compounds they produce themselves, as outlined above. Therefore, if an isolate is resistant to an antimicrobial produced by another strain, it can be deduced that both produce the same compound. Our findings showed that the marine isolates may be capable of producing one of a number of known *Bacillus* bacteriocins (lichenicidin, haloduracin, megacin or cerein), as they were all resistant to CFS from strains known to produce these. However, this is not definitive, as *Bacillus* are capable of producing more than one bacteriocin; for example, *Bacillus subtilis* ATCC 6633 produces several bacteriocins [6]. Furthermore, the opposite was not true, *i.e.*, the known bacteriocin-producing *Bacillus* were for the most part sensitive to the compounds produced by the marine isolates. 

Cross sensitivity assays in conjunction with spectral and physicochemical data also revealed that a range of antimicrobial compounds were produced by the marine bacteria. All of the marine isolates were sensitive to the compounds produced by *B. licheniformis* WIT 564 and 570, suggesting that these two isolates produce compounds that differ from those produced by the others. *B. pumilus* WIT 560 and WIT 561 displayed almost identical spectra of inhibition, both against the other marine isolates and unrelated strains, and the compounds produced had almost identical heat, pH and enzyme sensitivity patterns, confirming PFGE findings that they are the same strain. Similarities were also observed for both the spectra of inhibition and physicochemical properties of the compounds produced by *B. licheniformis* WIT 568 and 569. While *B. pumilus* WIT 571 and WIT 572 displayed the same spectra of inhibition against the other *Bacillus*, their activity against other genera as well as their physicochemical properties demonstrate that they produce different compounds. 

However, interpretation of spectral and physicochemical data is complicated by the fact that the marine isolates may produce more than one bacteriocin. As a result, a molecular approach was employed and three of the *B. licheniformis* isolates were found to harbor the gene encoding one of the peptides of the two-peptide bacteriocin lichenicidin. These isolates were then selected for a proof-of-concept exercise whereby lichenicidin production was thoroughly investigated and confirmed. Lichenicidin is a lantibiotic (a post-translationally modified bacteriocin with unusual lanthionine and/or β-methyllanthionine residues [4]). Its production has previously been reported in other *B. licheniformis* strains, namely ATCC 14580/DSM 13 [12,18] as well as VK21 [19] and I89 [20], both of which were isolated from hot springs, demonstrating that it is not unique to the marine environment. The lichenicidin peptides produced by the marine isolates were active only in combination, as previously observed for the hot spring isolates [19,21]. Conversely, those produced by *B. licheniformis* ATCC 14580 were active when tested alone as well as in combination, confirming previous findings for this strain [12]. This may reflect differences in the peptide concentration produced by different isolates. Moreover, this is the first report of lichenicidin production by a marine bacterium and the first definitive proof that any bacteriocin is produced by an algal-associated bacterium. Indeed, only a limited number of marine bacteriocins have been discovered to date. Many of these are produced by bacteria from marine animals, mainly fish, as recently reviewed by Desriac *et al.* [7]. Some examples include enterocin P produced by *E. faecium* isolated from turbot and nisin F and vibriocin AVP10 produced by catfish isolates of *Lc. lactis* and *Listonella anguillarum,* respectively [22]. Furthermore, although *Bacillus* spp. are well recognized as bacteriocin producers [6], no other bacteriocins have been isolated from marine-derived *Bacillus*, to date, with the exception of one from a *B. cereus* strain of fish origin [23]. Others produced by *B. licheniformis* strains of fish [24] and seaweed [25] origin can only be referred to as bacteriocin-like, due to a lack of characterization. 

However, while three of the marine *B. licheniformis* from the present study harbor one of the lichenicidin-associated genes and produce active lantibiotic, the antimicrobial spectrum of their CFS differs. Furthermore, one of the lichenicidin producers is sensitive to the CFS from the other two, suggesting that they may each produce additional antimicrobial compounds. The physicochemical properties of the CFS of these strains also differ and are distinct from that of lichenicidin, as lichenicidin remains active following autoclaving and treatment with α-chymotrypsin and trypsin [18], whereas the antimicrobials released from the cells of the marine *Bacillus* lost activity above 60 °C, and those from WIT 562 and 564 lost some activity after incubation with trypsin. However, lichenicidin may not be released into the supernatant, in agreement with the findings of Begley *et al.* [12] and Dischinger *et al.* [18] (although conversely, Caetano *et al.* [21] and Shenkarev *et al.* [19] did find it in the CFS). Notably, Dischinger *et al.* [18] reported that *B. licheniformis* DSM 13 produced a second antimicrobial compound in addition to lichenicidin and found that this compound was released into the CFS. Taken together, these data suggest that the lichenicidin producers may each produce additional antimicrobial compounds, a phenomenon which is not unusual for *Bacillus* [6]. In that case, separation of the antimicrobials released into the supernatant would be required in order to obtain more meaningful data on compound characteristics. 

While PCR screening was successful in identifying three of the marine bacteria as lichenicidin producers, the remainder of the isolates did not harbor structural genes for any of the known *Bacillus* bacteriocins for which PCR primers could be designed. These negative PCR outcomes suggest strongly that these isolates produce novel bacteriocins, as supported by data from cross sensitivity assays with known bacteriocin-producing strains. Their physicochemical properties and inhibitory spectra also suggest that they are distinct from other *Bacillus* bacteriocins for which PCR primers could not be designed. This comparison is not exhaustive due to the range of *Bacillus* bacteriocins discovered to date [6] but the bacteriocins uncovered in this study differ from the few discovered to date from marine *Bacillus*. For example, even though the compound(s) produced by *B. licheniformis* WIT 565 and 566 resemble a bacteriocin-like compound produced by a *B. licheniformis* strain of fish origin (e.g., resistance to trypsin, sensitivity to pronase E), they are less resistant to thermal treatment. Two of the marine-derived *B.licheniformis* from the present study, WIT 567 and 568, produce antimicrobials with the same protease sensitivity as that of a bacteriocin-like compound derived from a *B. licheniformis* isolate from seaweed but were more resistant to acidic conditions [25]. The bacteriocins produced by *B. licheniformis* WIT 567 and 569 display similarities to bacillocin 490 [26] (although this does not have anti-staphylococcal activity), lichenin [27], a *B. licheniformis* bacteriocin [28] as well as that produced by a *B. cereus* isolate of fish origin [23] and a bacteriocin-like peptide produced by a soil-derived *B. licheniformis* [29]. They are sensitive to the same proteases and, like the previously described compounds, remain active at high temperatures and low pH. However, the bacteriocins from the present study resist autoclaving while the latter two are sensitive and the others have not been tested. The properties of the unidentified bacteriocins from the present study are also different to those of previously described bacteriocins produced by other *B. licheniformis* and *pumilus* from sources other than the marine environment; for example, pumicilin 4 [30] and those produced by *B. licheniformis* T6-5 [31]. However, additional studies are required to confirm identity of the bacteriocins and/or determine if they are novel.

## 4. Experimental Section

### 4.1. Bacterial Strains and Culture Conditions and Chemicals

All chemicals were purchased from Sigma Aldrich (Dublin, Ireland) unless otherwise stated. Bacterial strains used as indicators for antimicrobial characterization and as positive controls for bacteriocin production and their respective growth conditions are listed in the table in Table S3.

### 4.2. Collection of Seaweed, Sand and Seawater Samples

Samples of seven macroalgae, namely *Ascophylum nodosum*, *F. serratus*, *F. vesiculosus*, *P. palmata*, *P. lanosa*, *U. lactuca* and *Ulva* spp. were collected during low tide from October 2009 to April 2010 from two locations in Fethard Bay, Co. Wexford, on the south east coast of Ireland (N52°10′, W06°50′ and N52°11.5′, W06°49.3′). One sample of seawater and one sample of sand were also collected from one from these locations on one occasion (March 2010). Samples were stored in the dark during transport to the laboratory and were processed within 2 h of sampling.

### 4.3. Detection and Isolation of Bacteriocin-Producing Marine Bacteria

Approximately 10 g of each seaweed or sand sample was washed twice in sterile artificial seawater [3.33% (w/v) Instant Ocean Salt, Aquarium Systems, Sarrebourg, France] for 2 min at 100 rpm to remove loosely attached bacteria and debris. Samples were then homogenized as 10-fold dilutions in sterile artificial seawater for 4 min in a stomacher (Masticator, IUL Instruments, Barcelona, Spain). Ten-fold serial dilutions of these homogenates and the seawater sample were then prepared in artificial seawater and appropriate dilutions were spread-plated on the following media: (1) MA [Difco marine broth 2216 (Becton, Dickinson and Company (BD), Franklin Lakes, USA) with 1.5% (w/v) agar (Oxoid, Basingstroke, Hampshire, UK)], (2) Gram-negative isolation agar (GNA) [MA with 100 µg/mL novobiocin], (3) LNA [3.33% (w/v) Instant Ocean Salt, 0.05% (w/v) tryptone (BD), 0.005% (w/v) yeast extract (Merck, Darmstadt, Germany), 0.01% (w/v) β-glycerol phosphate disodium salt, 1.5% (w/v) agar], (4) LPMA [0.5% (w/v) peptone (BD), 0.1% (w/v) yeast extract, 3.33% (w/v) Instant Ocean Salt, 0.01% (w/v) ferric citrate, 1.5% (w/v) agar], (5) AIA and (6) starch yeast peptone seawater agar (SYP-SW) [32]. Nystatin (50 units/mL) was added to all media to inhibit the growth of yeasts and molds. Spore-forming bacteria were also isolated by heating the seaweed homogenates for 15 min at 80 °C prior to spread-plating on MA. Five sets of spread plates were prepared in this way on each medium (except for GNA for which three sets were prepared) so that each set could be overlaid with a different indicator strain, as outlined below. All plates were incubated at 28 °C for up to 6 weeks, during which they were checked periodically for growth. 

Two approaches were used to detect and isolate bacteriocin-producing bacteria. In the first approach, for each sample up to 10 colonies with different morphologies were picked from each medium (except for the MA on which spore-forming bacteria were selected) and re-streaked at least twice onto the corresponding medium to obtain pure cultures. Colony morphologies were noted and isolates were stocked at −20 °C in marine broth supplemented with 40% (v/v) glycerol. These isolates were subsequently screened for antimicrobial activity using the deferred antagonism assay outlined below.

In the second approach, plates with <300 colonies, from which colonies were not selected in the first approach, were overlaid with soft BHI agar (0.75% w/v agar) seeded with a 0.25% inoculum of an overnight culture of either *S. aureus* DPC 5246, *S.* Typhimurium LT2, *L. innocua* WIT 361 or *E. coli* DSM 10720 or a 0.75% inoculum of an overnight culture of *B. subtilis* ATCC 6633. Plates were incubated overnight at 37 °C. Any colony which was surrounded by a clear zone of inhibition was picked off from under the agar overlay and inoculated into marine broth to kill off any contaminating indicator bacteria. Following overnight incubation at 28 °C, these cultures were then streaked in duplicate onto MA. One plate was overlaid with the same indicator strain against which inhibitory activity was initially observed. If activity was confirmed, the isolate was re-streaked onto MA from the duplicate plate and stocked at −20 °C in marine broth supplemented with 40% (v/v) glycerol. Antimicrobial production was then confirmed using the deferred antagonism assay outlined below.

### 4.4. Deferred Antagonism Assays

The deferred antagonism assay was used to screen marine bacteria isolated using the first approach outlined above for antimicrobial production and also to confirm antimicrobial production in bacteria recovered using the second approach. For this assay, the marine isolates were grown overnight at 28 °C in marine broth agitated at 200 rpm. Five microliters of each culture was spotted onto MA and incubated at 28 °C until culture spots were approximately 0.5–1 cm in diameter. A number of sets of plates were prepared in this way and each set was overlaid with soft BHI agar seeded with one of the five indicator strains, as outlined above, as well as *Lc. lactis* HP, *Lb. bulgaricus* LMG 6901, *P. aeruginosa* PA01 or *L. monocytogenes* WIT 041 seeded into the appropriate soft agar (Table S3) at a rate of 0.25% (*Lb. bulgaricus*, *P. aeruginosa*, *L. monocytogenes*) or 0.5% (*Lc. lactis*). This assay was also used to test activity against *E. faecalis* ATCC 19433 and *C. sakazakii* ATCC 12868, except that the marine isolates were grown in BHI broth, spotted onto BHI agar and the *E. faecalis* indicator was seeded at a rate of 0.25% into soft MRS agar supplemented with 0.05% (w/v) L-cysteine hydrochloride. Any isolate producing a clear zone of inhibition against any of the indicators was investigated further. 

### 4.5. Well Diffusion Assays and Determination of Spectra of Inhibition and Cross Sensitivity

To determine if antimicrobial compounds were released into culture supernatants, CFS from marine isolates with confirmed antimicrobial activity were tested using the WDA, as described by Begley *et al.* [12] except that wells were 6 mm in diameter and CFS was obtained by centrifuging cultures at 18,620× *g* for 10 min, removing the supernatant and centrifuging again. The CFS was tested against all of the indicator strains listed in Table S3 (except for *B.megaterium*, *thuringiensis*, *licheniformis*, *halodurans*, *cereus and subtilis* A1/3, 168 and HIL Y85,54728) in order to determine the spectra of inhibition. All indicator strains were used at an inoculum of 0.25% (v/v) except for *Lc. lactis* (0.5%), *B. subtilis* (0.75%) and *Cl. difficile* (1.25%). Plates were incubated for 20 h at the appropriate temperature and examined for zones of inhibition. Zones, where present, were measured. Cross sensitivity assays were performed to test the marine isolates for activity against each other and also against known bacteriocin-producing strains (*B. licheniformis* ATCC 14580, *B. subtilis* ATCC 6633, *B. megaterium* 216, *B. cereus* CECT 5148, *B. halodurans* ATCC BAA-125D-5 and *Lc. lactis* NZ9700). Well diffusion assays were employed, using MA for marine isolates or BHI for bacteriocin-producing strains, both seeded with a 0.75% (v/v) inoculum of the test culture.

### 4.6. Sensitivity of Antimicrobial Compounds to Enzymes, Heat and pH

Sensitivity of the antimicrobial compounds to proteolytic enzymes and catalase was determined as outlined by O’Shea *et al.* [15] except that the enzyme solutions were 10 mg/mL (giving a final enzyme concentration of 5 mg/mL), protease Type I and pronase E were also used and enzymatic treatments were performed at 37 °C for 2 h. *Lc. lactis* NZ 9700 CFS was used as a positive control and *Lc. lactis* HP was used as the indicator organism. The heat stability of each antimicrobial was determined by assaying the antimicrobial activity of CFS by WDA following heating to 40, 50, 60, 70, 80, 90 or 100 °C for 30 min in a heating block and to 121 °C for 15 min in an autoclave. Sensitivity to pH was investigated by adjusting the pH of the CFS to 2, 3, 5, 7, 9, 11 or 12, incubating at 28 °C for 2 h and assessing activity using the WDA. For all of the above assays, antimicrobial activity was expressed in arbitrary units (AU) as defined by Ryan *et al.* [33] and the percentage of residual activity was calculated, where 100% activity was the activity measured prior to treatment. 

### 4.7. Effect of Growth Medium on Antimicrobial Production

Antimicrobial-producing marine isolates were cultured overnight at 28 °C and agitated at 200 rpm in the following media: BHI, Difco Marine broth 2216, Luria-Bertani (LB) broth (Merck), tryptic soy broth (BD), nutrient broth (Oxoid), *Bacillus* production medium [34] and Actinomycete isolation broth [0.2% (w/v) sodium caseinate, 0.01% (w/v) L-asparagine, 0.4% (w/v) sodium propionate, 0.05% (w/v) dipotassium phosphate, 0.01% (w/v) magnesium sulphate, 0.0001% (w/v) ferrous sulphate and 0.5% (v/v) glycerol]. Antimicrobial production was assessed by the WDA using *Lc. lactis* HP as the indicator organism.

### 4.8. Identification and Differentiation of Bacteriocin-Producing Marine Isolates

Marine isolates were initially characterized phenotypically by Gram staining, spore staining and oxidase and catalase tests. In addition, 16S rRNA gene sequencing was performed on each isolate, by amplifying the 16S rRNA gene in a 15 µL reaction volume using primers 63f and 1387r, purifying the PCR products and sequencing them on an AB 310 genetic analyzer (Applied Biosystems, CA, USA), as outlined by Coffey *et al.* [35]. Nucleotide sequences were compared with those in the GenBank database using the blast program [36] through the National Center for Biotechnology Information (NCBI) server [37]. TreeView 1.6.6 [38] was used to construct a phylogram from a ClustalW alignment (ClustalX) [39].

Molecular fingerprinting of the marine isolates was then performed by pulsed-field gel electrophoresis (PFGE), as described previously [40], using *Apa*I, *Xba*I and *Not*I. Electrophoresis conditions used were 200 V for 17 h with a pulse time from 1 to 15 s for *Apa*I; 10 h at 200 V with a pulse time from 1 to 15 s for *Xba*I and 15 h at 200 V with a pulse time from 1 to 12 s for *Not*I. A low-range molecular weight DNA marker (0.13–194.0 Kb; New England Biolabs, Beverly, MA) was used. 

### 4.9. Screening for Known Bacteriocin Genes and Sequencing of Lichenicidin Gene

PCR was used to screen the marine isolates for the presence of genes encoding known *Bacillus* bacteriocins, as well as general lantibiotic genes. Genomic DNA was extracted from overnight cultures using a DNeasy 96 blood and tissue kit (Qiagen, Crawley, UK). The PCR primers used are listed in Table 3 and were synthesized by Eurofins (MWG Operon, Ebersberg, Germany). Some were designed in the present study based on gene sequences associated with previously identified bacteriocins obtained from Genbank. Sequences of the other PCR primers were obtained from previous studies (Table 3). Annealing temperatures were optimized using a gradient thermal cycler (Applied Biosystems). PCR conditions were as follows; initial denaturation at 95 °C for 5 min, followed by 35 cycles of 95 °C for 1 min, appropriate annealing temperature (Table 3) for 1 min, 72 °C for 1 min, followed by a final extension at 72 °C for 5 min.

PCR products obtained using lichenicidin primers were firstly cleaned using a DNA Clean and Concentrator™-5 kit (Zymo Research, Irvine, CA, USA) and then cloned using a PCR cloning kit (Qiagen) as per the manufacturer’s instructions. Recombinant plasmids were purified using the GenElute™ plasmid miniprep kit, as per the manufacturer’s instructions. T7 promoter and M13 forward (−20) primers were used for the sequencing reactions of the cloned PCR products. DNA sequencing was performed as described by Coffey *et al.* [35] and the sequences obtained were analyzed using BLAST software [36] from the Genbank (NCBI) database. Nucleic acid and deduced amino acid sequences were analyzed with DNASTAR software (DNASTAR Inc., Madison, USA).

### 4.10. Extraction and Purification of Lichenicidin

Lichenicidin was extracted and purified from the marine isolates found to harbor the *bli04127* gene that encodes one of the lichenicidin peptides and *B. licheniformis* ATCC 14580 (known lichenicidin-producing strain) using solid phase extraction followed by reverse-phase HPLC, as described by Begley *et al.* [12] with the following modifications; the HPLC column was developed in a gradient of 25% to 60% acetonitrile-0.1% (v/v) trifluoroacetic acid at a flow rate of 2.5 mL/min. HPLC fractions were analyzed by matrix-assisted laser desorption ionization time of flight (MALDI TOF) mass spectrometry, as outlined by Begley *et al.* [12]. Fractions with masses corresponding to those previously determined for lichenicidin [12] were tested for antimicrobial activity (individually and in combination) against *Lc. lactis* HP using the WDA. 

## 5. Conclusions

The present study assessed the bacteriocinogenic potential of marine bacteria and successfully recovered a number of *Bacillus* isolates, mainly from seaweeds, but also sand and seawater, that produce a range of bacteriocins, one of which was identified as lichenicidin. To the best of our knowledge, this is the first study to conclusively show that algal-associated bacteria, on the one hand, and marine *Bacillus* spp. on the other, produce a bacteriocin, albeit one that has previously been identified and is not unique to the marine environment. The absence of genes for known bacteriocins, together with the physicochemical properties of the bacteriocins produced, strongly suggests that the other marine isolates produce novel bacteriocins. Overall, this study provides further evidence of the value of marine bacteria as a source of antimicrobial agents which could potentially be exploited in medical, food or animal feed applications. However, the tedious nature of function-based screening, together with the fact that bacteriocin-producers may well be overlooked, mean that a genomic approach may prove a more useful strategy for the discovery of novel marine-derived antimicrobial agents in future studies. 

## Supplementary Files

Supplementary File 1: PDF-Document (PDF, 227 KB)
