# Supplementary material for: Assessment of the Bacteriocinogenic Potential of Marine Bacteria Reveals Lichenicidin Production by Seaweed-Derived Bacillus spp
Source: Mar Drugs. 2012 Oct 18;10(10):2280–99. doi: 10.3390/md10102280 (PMC3497023; doi:10.3390/md10102280)
Supplement: Supplementary File 1: — PDF-Document (PDF, 227 KB) [file marinedrugs-10-02280-s001.pdf]

Supplementary Information

Figure S1. Phylogenetic tree of antimicrobial-producing marine bacteria based on comparison of partial 16S rRNA gene sequences using the neighbor-joining method. The tree includes strain relatives to isolates determined by BLAST search. Out grouping was performed with *Clostridium.* [a] Marine isolates subsequently shown to produce lichenicidin as well as a known lichenicidin producer are shown in bold; [b] Denotes isolates with identical genetic fingerprints.

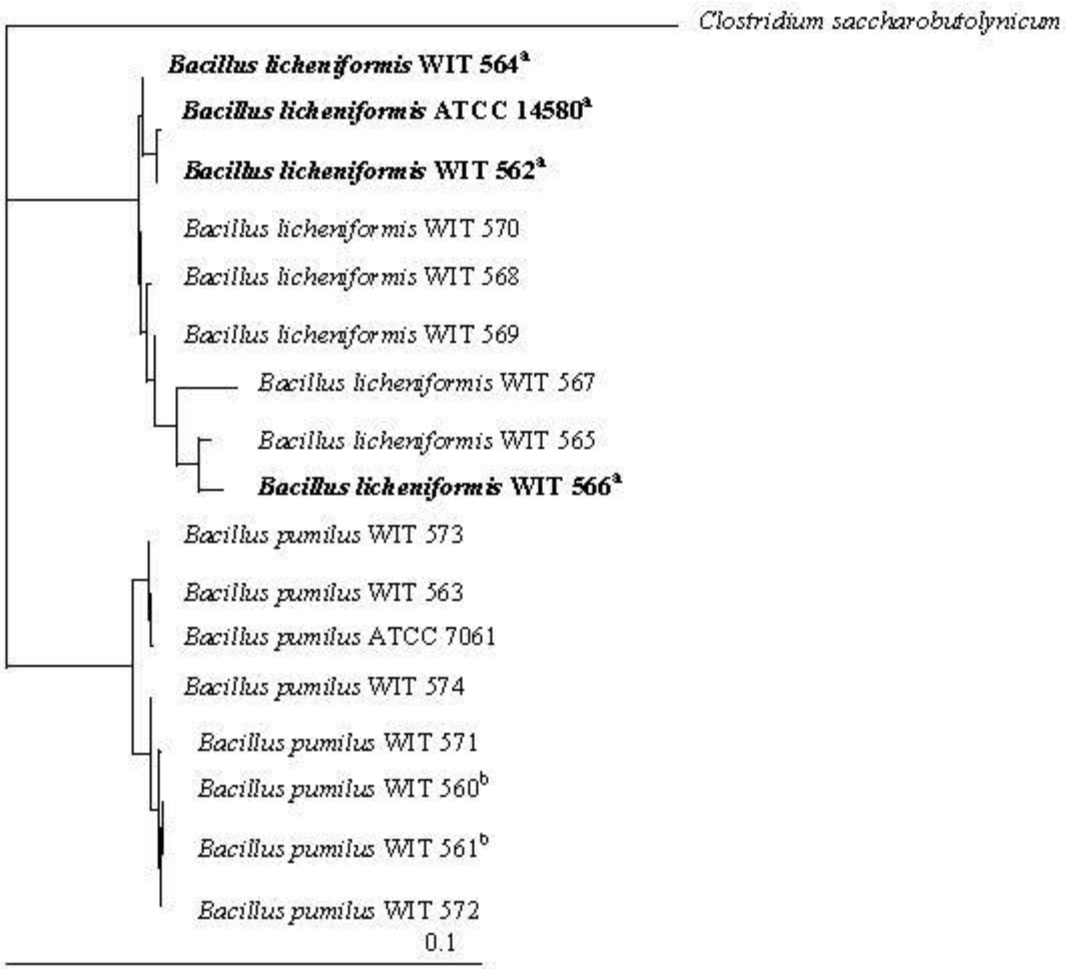

Figure S2. Representative PFGE patterns of *Not*I-digested genomic DNA from antimicrobial-producing *Bacillus* isolates. M: Low-range molecular weight marker; lane 1: WIT 560; lane 2: WIT 561; lane 3: WIT 562; lane 4: WIT 563; lane 5: WIT 564; lane 6: WIT 565; lane 7: WIT 566; lane 8: WIT 567; lane 9: WIT 568, lane 10: WIT 569; lane 11: WIT 570; lane 12: WIT 571; lane 13: WIT 572; lane 14: WIT 573, lane 15: WIT 574. [a] Denotes isolates with identical genetic fingerprints; [b] Isolates subsequently shown to produce lichenicidin.

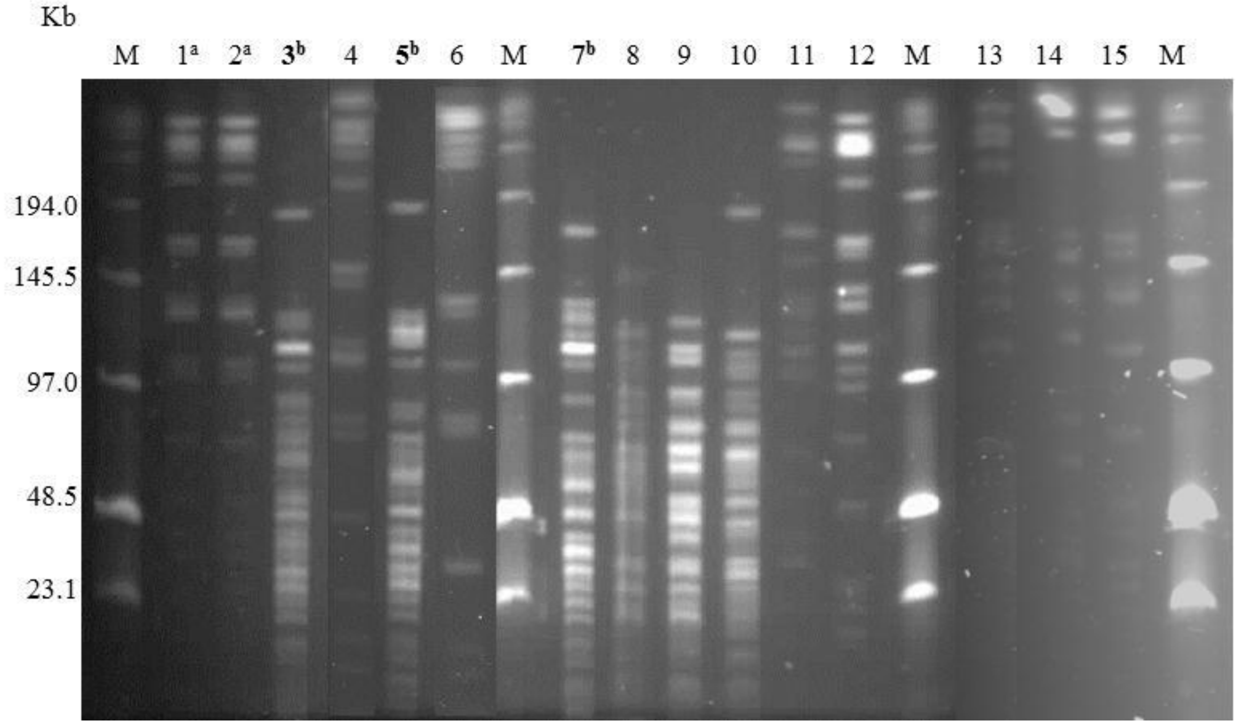

Table S1. Antimicrobial activity of marine isolates cultured using a range of different growth media [a].

Test Strain	BHI broth	Luria Bertani broth	Marine broth	Nutrient broth	*Bacillus* production medium	Tryptic soy broth	Actinomycete isolation broth
**WIT 562** [b]	+	+	++++	++++	++++	+	++++
**WIT 564** [b]	++	++	++++	++++	++++	++	+++
**WIT 566** [b]	+	+++	++++	++++	++++	-	+
WIT 560 [c]	++	+	++++	+++	+++	+	-
WIT 561 [c]	++	-	+++	+++	+++	+	-
WIT 563	+++	-	+++	+++	+	+	-
WIT 565	++	++	++++	++++	++++	-	-
WIT 567	++	++	++++	+++	++++	++	++
WIT 568	++++	++++	++++	++++	++++	++++	+++
WIT 569	+++	+++	++++	++++	++++	+++	++
WIT 570	++++	++++	++++	++++	++++	++++	++++
WIT 571	++	-	++	++	++	++	-
WIT 572	++	-	++++	+++	+++	-	-
WIT 573	+	-	++	+++	++	+	-
WIT 574	++	+	++	++	++	+	-

[a] Mean radii of zones of inhibition from cell-free supernatant assayed against *L. lactis* HP or *Lb. bulgaricus* LMG 6901 in triplicate well diffusion assays. + = 0.1–1 mm, ++ = 1.1–2 mm, +++ = 2.1–3 mm, ++++ > 3 mm; - = no antimicrobial activity; [b] Isolates subsequently shown to produce lichenicidin are grouped together and shown in bold; [c] Denotes isolates with identical genetic fingerprints.

Table S2. Cross-sensitivity assay of antimicrobial-producing marine isolates against each other and known bacteriocin-producing strains [a].

Test strain	WIT 560	WIT 561	WIT 562	WIT 563	WIT 564	WIT 565	WIT 566	WIT 567	WIT 568	WIT 569	WIT 570	WIT 571	WIT 572	WIT 573	WIT 574	*B. subtilis* [b]	*B. licheniformis* [c]	*B. halodurans* [d]	*B. megaterium* [e]	*B. cereus* [f]
**WIT 562** [g]	-	-	-	-	-	-	-	-	-	-	-	-	-	-	+	-	-	+++	++	-
**WIT 564** [g]	+++	+++	+++	++++	-	++++	++++	++	++	++	+++	+++	+++	+++	+++	+	++	++++	+++	-
**WIT 566** [g]	++	++	-	-	-	-	-	-	-	+	-	+	+	+	+	+	-	++++	++	-
WIT 560 [h]	-	-	++	+	-	-	-	++	-	-	+	+	-	+	+	-	-	++	-	-
WIT 561 [h]	-	-	++	+	-	-	-	++	-	-	+	-	-	+	-	-	-	++	-	-
WIT 563	-	-	++	-	-	-	-	++	-	-	-	+	+	+	-	-	-	+++	-	-
WIT 565	++	++	-	-	-	-	-	-	-	+	-	+	++	+	+	+	-	++	-	-
WIT 567	-	-	-	-	-	-	-	-	-	-	-	-	-	-	-	+	++	+++	++	-
WIT 568	+++	+++	+	+++	++	++	++	-	-	-	+++	+++	+++	+++	+++	+	++	++++	++	-
WIT 569	+++	+++	+	+++	++	+++	++	-	-	-	+++	+++	+++	+++	+++	+	++	++++	++	-
WIT 570	++++	++++	+++	++++	+	+++	+	++	+	+	-	++++	++	++++	++	-	++	+++	+++	+
WIT 571	-	-	++	+	-	-	-	++	-	-	+	-	-	+	-	-	-	++	-	-
WIT 572	-	-	++	+	-	-	-	++	-	-	+	-	-	+	-	-	-	++	-	-
WIT 573	-	-	-	-	-	-	-	+	-	-	-	-	-	-	-	-	-	++++	-	-
WIT 574	-	-	-	+	-	-	-	-	-	-	-	-	-	+	-	-	-	+	-	-
B. subtilis [b]	+++	+++	-	+++	-	+++	-	-	-	-	+++	++	+++	++	+++	-	+	++++	++++	+
B. licheniformis [c]	-	-	-	-	-	-	-	-	-	-	-	-	-	-	-	-	-	-	-	-
B. halodurans [d]	-	-	-	-	-	-	-	-	-	-	-	-	-	-	-	-	-	-	-	-
B. megaterium [e]	-	-	-	-	-	-	-	-	-	-	-	-	-	-	-	-	-	-	-	-
B. cereus [f]	-	-	-	-	-	-	-	-	-	-	-	-	-	-	-	-	+	++++	-	-
Lc. lactis NZ 9700 [i]	++	++	++	++	++	++	+++	+	+	++	++	++	+	++	++	+	ND	ND	ND	ND

[a] Mean radii of zones of inhibition from cell-free supernatant analyzed in triplicate in well diffusion assays. + = 0.1–1 mm, ++ = 1.1–2 mm, +++ = 2.1–3 mm, ++++ > 3 mm; - = no antimicrobial activity. ND = not determined; [b] ATCC 6633 (subtilin producer); [c] ATCC 14580 (lichenicidin producer); [d] ATCC BAA-125D-5 (haloduracin producer); [e] 216 (megacin A-216 producer); [f] CECT 5148 (cerein 7B producer); [g] Isolates subsequently shown to produce lichenicidin are grouped together and shown in bold; [h] Denotes isolates with identical genetic fingerprints; [i] *Lc. lactis* NZ 9700 (nisin producer).

Table S3. Bacterial strains used in this study as indicators for antimicrobial characterization and as positive controls for bacteriocin production and their growth conditions.

Bacterial strain	Use in this study	Growth medium	Incubation temperature (°C)	Growth conditions
Listeria innocua WIT 361	Indicator	BHI [a]	37	Aerobic
L. monocytogenes WIT 041	Indicator	BHI	37	Aerobic
Staphylococcus aureus DPC 5246	Indicator	BHI	37	Aerobic
Methicillin resistant *S. aureus* (MRSA) W73365	Indicator	BHI	37	Aerobic
Lactococcus lactis HP	Indicator	LM17 [b]	28	Aerobic
Lc. lactis NZ 9700	Positive control (nisin producer)	GM17 [c]	28	Aerobic
Lc. lactis DPC 3147	Positive control (lacticin 3147 producer)	GM17	28	Aerobic
Enterococcus faecium ATCC 19434	Indicator	MRS + cysteine [d]	37	Aerobic
Enterococcus faecalis ATCC 19433	Indicator	MRS + cysteine	37	Aerobic
Lactobacillus bulgaricus LMG 6901	Indicator	MRS + cysteine	37	Anaerobic
Clostridium difficile ATCC 43593	Indicator	RCM [e]	37	Anaerobic
Bacillus halodurans ATCC BAA-125D-5	Indicator, positive control (haloduracin A1 & A2 producer)	BHI	37	Aerobic, 200 rpm
Bacillus cereus CECT 5148	Indicator, positive control (cerein 7B producer)	BHI	37	Aerobic, 200 rpm
Bacillus licheniformis ATCC 14580	Indicator, positive control (lichenicidin producer)	BHI	37	Aerobic, 200 rpm
Bacillus megaterium 216	Indicator, positive control (megacin A-216 producer)	BHI	37	Aerobic, 200 rpm
Bacillus subtilis ATCC 6633	Indicator, positive control (subtilin and subtilosin producer)	BHI	37	Aerobic, 200 rpm [f]
B. subtilis A1/3	Indicator, positive control (ericin A producer)	BHI	37	Aerobic, 200 rpm
B. subtilis 168 (ATCC 23857)	Indicator, positive control (sublancin producer)	BHI	30	Aerobic, 200 rpm
B. subtilis HIL Y85,54728	Indicator, positive control (mersacidin producer)	BHI	37	Aerobic, 200 rpm
Bacillus thuringiensis NEB 17	Indicator, positive control (thuricin 17 producer)	BHI	28	Aerobic, 200 rpm
E. coli DSM 10720	Indicator	BHI	37	Aerobic
Salmonella Typhimurium LT2	Indicator	BHI	37	Aerobic
Pseudomonas aeruginosa PA01	Indicator	BHI	28	Aerobic
Cronobacter sakazakii ATCC 12868	Indicator	BHI	37	Aerobic

[a] Brain heart infusion (BHI; Oxoid, Basingstroke, Hampshire, UK); [b] LM17 [M17 (Oxoid) containing 0.5% (w/v) lactose]; [c] GM17 [M17 containing 0.5% (w/v) glucose]; [d] de Man, Rogosa and Sharpe (MRS) [(Becton, Dickinson and Company (BD), Franklin Lakes, USA] supplemented with 0.05% (w/v) L-cysteine; [e] Reinforced clostridial medium (RCM; Merck, Darmstadt, Germany); [f] Broths were incubated with shaking at 200 rpm.
